# An antibacterial and absorbable silk-based fixation material with impressive mechanical properties and biocompatibility

**DOI:** 10.1038/srep37418

**Published:** 2016-11-21

**Authors:** Chenglong Shi, Xiaobing Pu, Guan Zheng, Xinglong Feng, Xuan Yang, Baoliang Zhang, Yu Zhang, Qingshui Yin, Hong Xia

**Affiliations:** 1 Southern Medical University, Guangzhou, China; 2 Department of Orthopedics, Guangzhou General Hospital of Guangzhou Military Command, Guangzhou, China; 3 No. 188 hospital of People’s Liberation Army, Chaozhou, China; 4 Department of Orthopedics, Sun Yat-sen Memorial Hospital, Sun Yat-sen University, Guangzhou, China; 5 The Central Hospital of Yong Zhou, Yongzhou, China

## Abstract

Implant-associated infections and non-absorbing materials are two important reasons for a second surgical procedure to remove internal fixation devices after an orthopedic internal fixation surgery. The objective of this study was to produce an antibacterial and absorbable fixation screw by adding gentamicin to silk-based materials. The antibacterial activity was assessed against *Staphylococcus aureus (S. aureus*) and *Escherichia coli (E. coli*) *in vitro* by plate cultivation and scanning electron microscopy (SEM). We also investigated the properties, such as the mechanical features, swelling properties, biocompatibility and degradation, of gentamicin-loaded silk-based screws (GSS) *in vitro*. The GSS showed significant bactericidal effects against *S. aureus* and *E. coli*. The antibacterial activity remained high even after 4 weeks of immersion in protease solution. In addition, the GSS maintained the remarkable mechanical properties and excellent biocompatibility of pure silk-based screws (PSS). Interestingly, after gentamicin incorporation, the degradation rate and water-absorbing capacity increased and decreased, respectively. These GSS provide both impressive material properties and antibacterial activity and have great potential for use in orthopedic implants to reduce the incidence of second surgeries.

The use of internal fixation devices, especially metallic materials, is widespread and expected to increase in the future due to an aging population and improving medical care. However, second surgical procedures to remove internal fixation devices are common. According to a Finnish study, approximately 5% of all orthopedic procedures in the United States and nearly 81% of implants inserted for fracture fixation are removed after fracture healing, which greatly increases the pain and financial burden for patients^1^,^2^,^3^. The disadvantages of rigid plate and screw fixation, including infection, exposure, retained implants and pain, can be responsible for a second surgery^3^,^4^,^5^,^6^,^7^,^8^. With the development and use since the 1990 s of resorbable fixation devices of differing polymer compositions, including poly-L-lactic acid (PLLA), polyglycolic acid (PGA) and poly-lactic-co-glycolic acid (PLGA), numerous potential advantages have been shown to decrease the necessity for hardware removal and the risk of growth restriction and transcranial migration^4^,^9^,^10^. However, identified complications of resorbable fixation devices include infections^11^,^12^, self-limited local inflammatory reactions^13^,^14^,^15^, palpability^12^ and device removal^12^,^16^. Campbell *et al.* reported that the incidence of infection ranges from 0.2 to 2%, the incidence of inflammation reactions ranges from 0.7 to 14%, and the incidences of all causes of plate or screw removal range from 0.3 to 4%^4^. Mäkinen *et al.* explored a bioabsorbable ciprofloxacin-containing bone screw (Ab-PLGA) to prevent biomaterial-related infection^17^, but its lower strength and the abovementioned disadvantages have restricted the widespread clinical application of these resorbable implants. Low-load periarticular fractures, such as craniofacial fractures^11^,^18^,^19^, calcaneal fractures^9^ and metacarpal fractures^20^, remain the best indications for their use.

Recently, Perrone *et al.* reported that silk-based screws may become a new option for resorbable fixation devices that overcome the limitations of conventional resorbable materials due to their impressive mechanical features, biocompatibility, ability to promote bone remodeling and environmental stability^21^. In addition, the potential utility of easy implantation, autoclave sterilization and FDA-approved uses benefit their application as a medical device^21^,^22^. However, like all biomaterials, silk-based screws may carry an elevated risk of implant-associated infections because of the formation of bacterial biofilms^23^,^24^,^25^,^26^. Our research indicated that silk fibroin partly promoted the growth of *Staphylococcus aureus (S. aureus*), which may be the major disadvantage of the use of these screws as a fixation device. Antibacterial and bioabsorbable properties are indispensable for an ideal fixation device. The paramount importance of infection prevention should be emphasized because implant-associated infections are difficult to cure due to bacterial biofilms, and in many cases, removal is the only remedy^27^. Several biomaterial surface modifications have been proposed to impart silk fibroin with antibacterial properties, such as combination with chitosan^28^, chemical attachment of antimicrobial peptides^29^ and loading with silver nanoparticles^30^,^31^. However, in clinical applications, internal fixation materials must be autoclaved for sterilization, and high temperatures may affect the antibacterial properties of antimicrobial peptides^29^,^32^. Silk-based fixation materials will remain in the body for a long time, even for more than a year, but the safety of the long-term and continuous release of silver nanoparticles in the body is unclear^33^. Furthermore, internal fixation materials require a specific material form and mechanical strength, so these modifications may be unsuitable for silk-based fixation materials.

Thus, we explored the preparation of antibacterial silk-based screws from the incorporation of an antibacterial agent, gentamicin, to provide high and durable antimicrobial activity. In the present work, the properties of the silk-based screws containing gentamicin, such as the mechanical features, swelling properties, biocompatibility and degradation, were tested *in vitro.* Furthermore, the antibacterial activity of gentamicin-loaded silk-based screws (GSS) was assessed against gram-positive *S. aureus* and gram-negative *Escherichia coli (E. coli*) *in vitro*. Finally, we expected to obtain a resorbable and antimicrobial fixation device with high mechanical strength and impressive biocompatibility to reduce the risk of second surgery caused by infection and other complications.

## Materials and Methods

### Materials

Cocoons of *Bombyx mori* were purchased from the Shandong Academy of Sericulture, China. Gentamicin sulfate was purchased from the National Institutes for Food and Drug Control, China. *S. aureus* (ATCC25923) and *E. coli* (ATCC25922) were obtained from the Guangdong Institute of Microbiology, China. Protease type XIV (approximately 4 units/mg; mixture of at least three caseinolytic activities and one aminopeptidase activity) from *Streptomyces griseus* was purchased from Sigma-Aldrich (St. Louis, MO, USA), and all analytical-grade reagents were purchased from Aladdin (Shanghai, China).

### Specimen preparation

#### Preparation of HFIP silk solutions

Silk solutions were prepared from *B. mori* cocoons according to a previously reported procedure^21^,^34^. *B. mori* cocoons were boiled for 30 min in aqueous 0.02 M Na_2_CO_3_ and then washed thoroughly with distilled water. This treatment was repeated three times to obtain pure silk fibroin. Then, the dried silk fibroin (10 g) was dissolved in 100 ml of a ternary solvent, CaCl_2_–CH_3_CH_2_OH–H_2_O (1:2:8 molar ratio)^35^, at 85 °C until total dissolution, followed by dialysis (MWCO 3,500, Biosharp, Hefei, China) against distilled water for 2 days. The solution was centrifuged for 2 × 20 min at 18,000 rpm. The final solution was frozen for more than 2 days and vacuum-dried at −20 °C until complete sublimation. Gentamicin sulfate was dissolved in 1,1,1,3,3,3-hexafluoro-2-propanol (HFIP), and solutions were prepared at four concentrations (40, 20, 15 and 10 mg/ml). For 20 w/v% silk in HFIP, 10 g of vacuum-dried silk was cut into small pieces and packed into 50 ml syringes, after which 40 ml of the prepared HFIP solution was added.

#### Preparation of silk blanks and screws

Silk screw blank molds (3.3 cm height, 0.8 cm diameter) were prepared using wax from MachinableWax.com (USA) and filled with the HFIP silk solution. The molds were placed in methanol for 3-4 days. Subsequently, a methanol-to-water gradient was applied, and water was added at 4 × 1 h intervals to slowly transition the solution from 100% methanol to 100% water. The materials were then immersed in distilled water for 2 days prior to drying and baking in a 60 °C oven. Parts of the silk blanks were cut into slices (length, 0.1 cm, and diameter, 0.5 cm) as the samples for the experiment, and other parts were machined into screws. Finally, four different concentrations of GSS and pure silk-based screws (PSS) were prepared: GSS1 (16 mg/g silk), GSS2 (8 mg/g silk), GSS3 (6 mg/g silk), GSS4 (4 mg/g silk) and PSS.

### Characterization of GSS/PSS

#### Surface characteristics

The surface morphology of the samples was examined by field-emission scanning electron microscopy (FE-SEM, Zeiss, Germany).

#### Mechanical properties

A three-point bending test and a double-shear test were performed using an ElectroForce^®^ 3500 machine (Bose Corporation, Eden Prairie, USA) at room temperature with five repetitions for each test. The materials were dried and made into rods with 17 mm length and 5 mm diameter for testing. The three-point bending test was carried out at a crosshead speed of 5 mm/min. The bending strength was calculated as follows: bending strength = 8PL/πd^3^, where P is the maximum load applied, L is the span length, and d is the rod diameter. The span length for the three-point bending test was 10 mm. The double-shear test was carried out with a testing speed of 5 mm/min. The shear strength was calculated as follows: shear strength = F/2 A, where F is the force at fracture and A is the cross-sectional area of the rod. In addition, the Young’s modulus of the materials was measured by the impulse excitation technique (IET) using a Grindosonic^®^ instrument (Lemmens Electronics, Leuven, Belgium) according to the manufacturer’s standard method (ASTM E1876-2009).

#### *In vitro* degradation

The slice samples (n = 3) were incubated at 37 °C in a solution containing 1 mg/ml protease XIV in phosphate-buffered saline (PBS) or PBS alone as the control. The solution was refreshed every 48 h. At designated time points (1, 2, 4, 8 and 12 weeks), the samples were washed thoroughly with PBS, dried, weighed and observed by SEM. The weight loss (%) was calculated from the following equation: 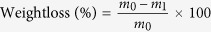, where *m*_0_ and *m*_1_ are the initial and final dry mass of the sample, respectively.

#### *In vitro* swelling properties

Before testing, the dry weight (W_d_) and diameter (D_d_) of silk blanks were obtained. Silk blanks (n = 6) were immersed in PBS at 37 °C for various lengths of time. At designated time points, the blanks were wiped with filter paper to remove any surface moisture. Subsequently, the wet weight (W_s_) and diameter (D_s_) of the sample were measured. The water uptake (%) and increase in diameter (%) were calculated from the following equation: 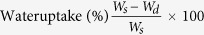; increase in diameter (%) = 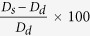.

### Effects of PSS/GSS on the proliferation and apoptosis of MC3T3-E1 *in vitro*

#### Cell proliferation

According to ISO 10993-12, a sample with 0.5 cm diameter and 0.1 cm thickness was immersed in 0.18 ml (3 cm^2^/ml) of alpha-Minimum Essential Medium (α-MEM, Gibco, Grand Island, NY, USA) supplemented with 10% fetal bovine serum (FBS, HyClone, Smithfield, Australia) for 24 h. MC3T3-E1 cells (Cell Bank of the Chinese Academy of Sciences, Shanghai, China) were seeded on 96-well plates at 4 × 10^4^ cells/well. After 24 h, the medium of the test group was replaced with the leaching liquor acquired from the samples or fresh α-MEM containing 10% FBS. After the desired period of time (1, 3 and 5 days), the medium was removed, and 150 μl of Cell Counting Kit-8 (CCK-8, Dojindo, Kumamoto, Japan) solution was added to each well, after which the solution was incubated in a 5% CO_2_ incubator at 37 °C for 1.5 h. The absorbance was measured at 450 nm using an automated ELISA microplate reader (Thermo, Multiskan GO, USA). The cell number was correlated with the optical density (OD).

#### Cell apoptosis

MC3T3-E1 cells were seeded on 24-well plates at a concentration of 10^5^ cells/ml. After 24 h, the medium was replaced with the leaching liquor acquired from the samples or fresh α-MEM containing 10% FBS or 5% dimethyl sulfoxide (DMSO, Sigma, St. Louis, MO, USA) as the positive control. Subsequently, the cells were cultured for 24 h, washed three times with PBS (Bioscience, Shanghai, China) and resuspended in 100 μl of α-MEM with 1% FBS. The cells were stained in a 96-well microplate with Guava Nexin Reagent (Millipore, Billerica, MA, USA) in a final volume of 200 μl. After 20 min of incubation at room temperature, the samples were analyzed on a Guava easyCyte 5HT flow cytometer (Millipore, USA), and the data were analyzed using Guava Nexin software, v2.2.2.

### *In vitro* antimicrobial activity assay

#### Inhibition zone

The antibacterial activity of the GSS and PSS samples was determined from the inhibition zone using *S. aureus* and *E. coli*. An aliquot of 100 μl of a bacterial suspension with a concentration of 10^7^ CFU/ml was seeded on a standard agar culture plate, and the sterilized samples were then placed on the plates. After 24 h of incubation, the diameter of the inhibition zone (mm) surrounding each sample was measured.

#### Bacterial counting and morphological observations

Sterilized samples of the GSS and PSS were placed on the bottom of the wells in sterilized 48-well plates. Then, a drop of bacterial suspension with a concentration of 10^7^ CFU/ml was added. A blank well was used as the control. After different incubation periods at 37 °C (15 min, 30 min, 1 h, 2 h, 6 h, 12 h and 24 h), the plates were placed in an ultrasonic washer (40 kHz) for 2 min to dislodge the bacteria retained on the samples. A total of 100 μl of the suspended substance was extracted from each well, and serial 10-fold dilutions were performed with physiological saline. Subsequently, three 100 μl drops of each dilution were introduced to a standard agar culture plate for further incubation for 24 h. The active bacteria were counted according to the National Standard of China GB/T 4789.2 protocol, and the antibacterial ratio (%) was used to quantitatively assay the antimicrobial activity of each sample in this work, which is defined as follows: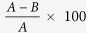, where A is the average number of bacteria on the control samples (CFU/sample) and B is the average number of bacteria on the testing samples (CFU/sample). The morphology of *S. aureus* and *E. coli* on samples after different incubation periods were observed by SEM as described above.

#### Antimicrobial durability

The antimicrobial durability was measured by immersing the samples in 1 mg/ml protease XIV for different durations (1, 2, 4 and 8 weeks) prior to the activity assay.

#### Release of gentamicin

Samples of GSS1 (n = 3) were immersed in PBS at 37 °C. At predetermined time points, the solution was extracted and replaced with freshly prepared solution. The concentration of gentamicin was determined using the o-phthaldialdehyde method^36^. The product of the reaction between gentamicin and o-phthaldialdehyde was read on a fluorophotometer (Thermo, Multiskan GO, USA) at an excitation wavelength of 340 nm and emission wavelength of 455 nm. The concentration was determined according to a gentamicin standard curve (R^2^ = 0.997).

### Statistical analysis

All experiments were conducted in at least triplicate, and the data obtained were expressed as the mean ± standard deviation. Statistical analysis of the data was performed by unpaired t-test or one-way analysis of variance (ANOVA) with Student-Newman-Keuls (SNK) post hoc test using SPSS 20.0 software (SPSS Inc., Chicago, IL, USA). Differences between groups of *p* < 0.05 and *p* < 0.01 were considered significant and highly significant, respectively.

## Results

### Characterization of PSS/GSS

#### Surface characteristics

**Figure 1:**
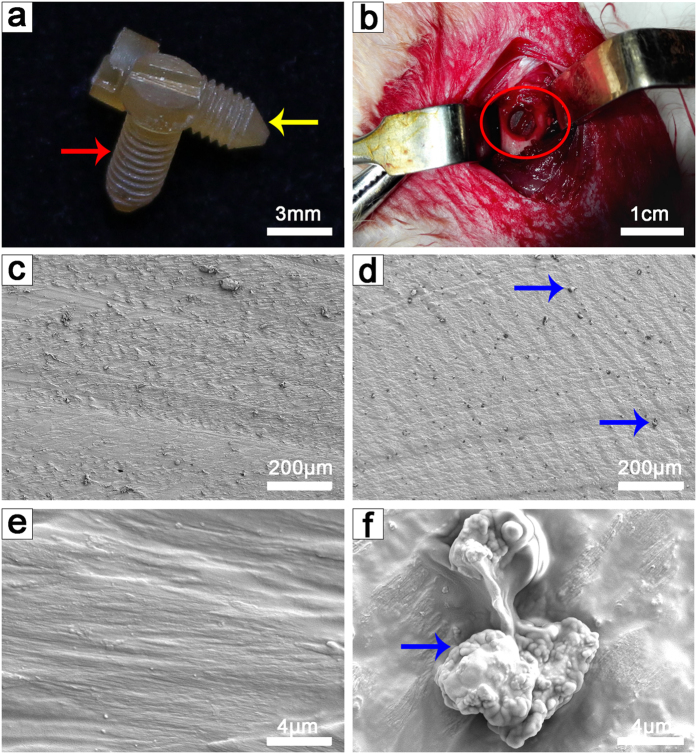
General images of the silk screws. (**a**) PSS (red arrow) and GSS (yellow arrow). The average diameter and length of the screws were 0.3 cm and 1 cm, respectively. (**b**) GSS implanted into a rabbit femur (red circle indicates GSS and femur). (**c**,**e**) SEM images of pure silk material after being cut into slices at various magnifications. (**d**,**f**) SEM images of gentamicin-loaded silk material after being cut into slices at various magnifications (blue arrows indicate gentamicin particles).

The dimensions of the screws after drying consisted of an average length of 1 cm and diameter of 0.3 cm (Fig. 1a), which were appropriate for implantation into a rabbit (2.5 kg) femur *in vivo* (Fig. 1b). SEM images showed a rough surface of the PSS slices (Fig. 1c,e). Figure 1d and f reveal that gentamicin particles differing in size from 3 to 15 μm were uniformly inlaid in the silk protein of the GSS samples.

In addition, the surface structure of the silk protein between PSS and GSS showed no obvious differences.

#### Mechanical properties

Table 1 lists the mechanical test data for the PSS and GSS samples. The PSS samples achieved a bending strength of 204.1 ± 9.1 MPa (n = 5) and shear strength of 35.6 ± 5.1 MPa (n = 5). Compared with PSS, the test data for GSS were not significantly different (*p *= 0.80 and 0.97) at 202.8 ± 7.8 MPa (n = 5) and 35.4 ± 6.1 MPa (n = 5), respectively. In addition, the Young’s modulus values of PSS and GSS were 8.3 ± 0.7 GPa and 8.5 ± 0.7 GPa, respectively. There was also no significant difference (*p *= 0.61) between GSS and PSS in terms of the Young’s modulus.

#### *In vitro* degradation

**Figure 2:**
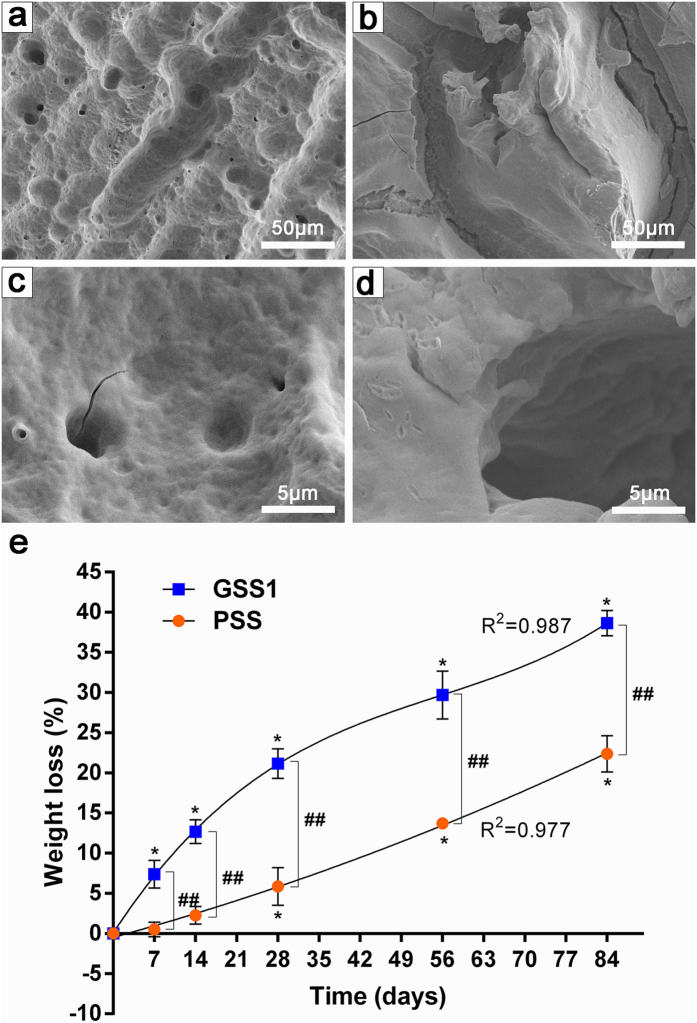
SEM images and weight loss of PSS and GSS1 after enzymatic degradation. (**a**,**c**) SEM images of PSS samples incubated in protease XIV for 12 weeks at various magnifications. (**b**,**d**) SEM images of GSS1 samples incubated in protease XIV for 12 weeks at various magnifications. (**e**) Weight loss (%) of samples as a function of time in 1 mg/ml protease XIV with curve fits. The weight losses of the PSS and GSS1 were consistent with a second-order polynomic trendline (y = 0.00087x^2^ + 0.2003x − 0.495, R^2^ = 0.98) and third-order polynomic trendline (y = 0.000096x^3^ − 0.016x^2^ + 1.121x + 0.0556, R^2^ = 0.99), respectively (n = 3, *indicates significant differences (*p *< 0.05) compared with the original samples, and ^##^indicates highly significant differences (*p *< 0.01) between PSS and GSS1).

The *in vitro* degradation of samples incubated in protease XIV, which has been shown to effectively degrade silk films and fibers^21^,^37^, was observed, with the goal of predicting the resorption of silk screws into the body. Surface erosion was observed by SEM on the PSS (Fig. 2a,c) and GSS1 (Fig. 2b,d) samples incubated for 12 weeks (84 days), indicating the occurrence of enzymatic degradation *in vitro* compared with the initial samples (Fig. 1). However, a number of voids were present in the surfaces of the GSS1 samples incubated for 12 weeks (Fig. 2b,d). As shown in (Fig. 2e), significant differences (*p* < 0.05) in the weight loss of the PSS were observed after 4 weeks (28 days) of incubation, with 22.4 ± 2.3% weight loss after 12 weeks. However, just 1 week (7 days) later, the weight loss of GSS1 was significantly different (*p* < 0.05) compared with the initial samples, and there was 38.7 ± 1.6% weight loss after 12 weeks (84 days). The weight losses of PSS and GSS1 were consistent, with second-order (R^2^ = 0.98) and third-order (R^2^ = 0.99) polynomic trendlines, respectively. All differences in weight loss (%) of the PSS and GSS1 samples were considered significant for all time points (*p* < 0.01), which indicate that the degradation rate of GSS1 is faster than that of PSS in protease type XIV. No significant differences in mass loss were observed in the PBS-incubated samples over time (Figure S1), which is shown in the supplementary information.

#### *In vitro* swelling properties

**Figure 3:**
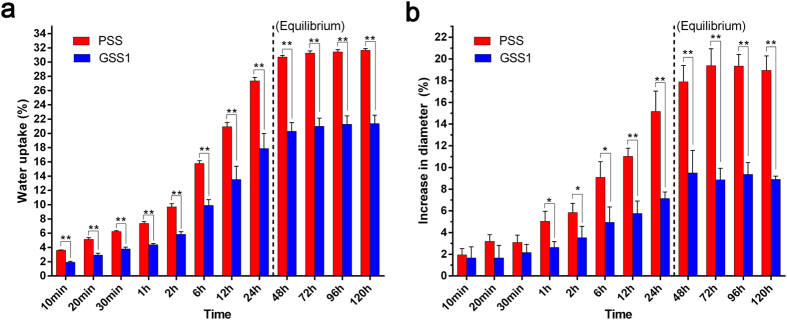
*In vitro* swelling properties. (**a**) Water uptake (%) of samples and (**b**) increase in diameter (%) of samples immersed in PBS for 0 min, 10 min, 20 min, 30 min, 1 h, 2 h, 6 h, 12 h, 24 h, 48 h, 72 h, 96 h and 120 h (n = 3, *p < 0.05 and **p < 0.01, NS indicates no significant differences using Dunnett’s multiple comparison test).

The water uptake and increase in diameter of the PSS and GSS1 *in vitro* are reported in (Fig. 3). The weight equilibrium water uptake values of the PSS and GSS1 after 48 h of hydration in PBS were 30.7 ± 0.3% and 20.3 ± 1.2%, respectively. Figure 3a shows a general decrease in the water uptake of GSS1 with time compared with the PSS (*p* < 0.01), signifying that GSS1 swells less and at a slower rate. In addition, the PSS gained a significant amount of weight after 10 min (*p* < 0.01), whereas the GSS did not gain a significant amount of weight until after 20 min of incubation (*p* > 0.05). At the same time, the weight equilibrium increases in the diameter of the PSS and GSS1 after 48 h were 17.9±1.5% and 9.5±1.1%, respectively. Figure 3b shows that the increase in the diameter of GSS1 was significantly smaller than that of PSS after 1 h (p < 0.05), which agrees with the results of water uptake. With respect to changes in the diameter of GSS1, no significant changes occurred between 0 and 30 min (p > 0.05), whereas no significant changes occurred between 0 and 10 min for PSS.

### Effects of PSS/GSS on the proliferation and apoptosis of MC3T3-E1 *in vitro*

**Figure 4:**
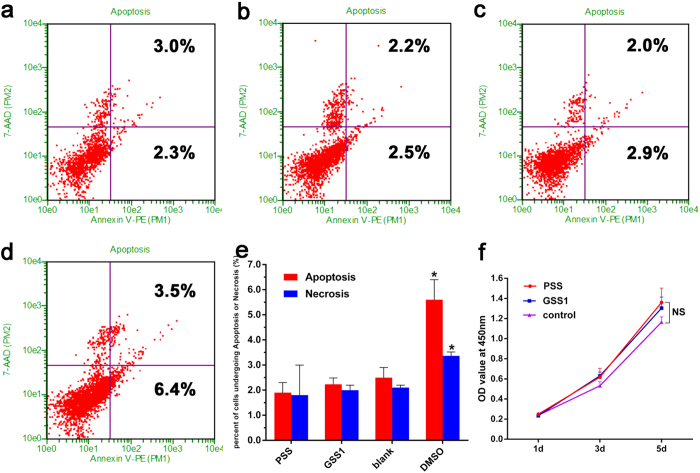
Cell apoptosis and proliferation assays with an Annexin V-FITC Apoptosis Detection Kit and a Cell Counting Kit-8. The percentage of Annexin V (+) 7-AAD (+) and Annexin V (+) 7-AAD (−) cells revealed necrosis and apoptosis, respectively. (**a**) PSS. (**b**) GSS1. (**c**) Blank control. (**d**) Positive control, DMSO. (**e**) Percentage of cells undergoing apoptosis or necrosis (%) in different groups. (**f**) Cell proliferation assay of GSS1 (n = 3, *p < 0.05, NS indicates no significant differences).

Because the ultimate objective of our work was to use this fixation device in clinical patients, the effects of the samples on *MC3T3-E1* proliferation and apoptosis were be assayed using the CCK-8 assay and flow cytometry. After 5 days of culturing, the OD values in the PSS and GSS1 groups were slightly higher than that in the blank control group (Fig. 4f), but no significant differences (*p *> 0.05) were observed. Additionally, the percentage of cells undergoing apoptosis and necrosis in the presence of PSS or GSS1 exhibited no significant differences compared with the blank control group but were lower significantly (*p *< 0.05) compared with the positive control group (Fig. 4e). The above results indicate that there were no detrimental effects to cell proliferation or apoptosis resulting from the PSS and GSS1.

### Antibacterial ability *in vitro*

#### Inhibition zone

**Figure 5:**
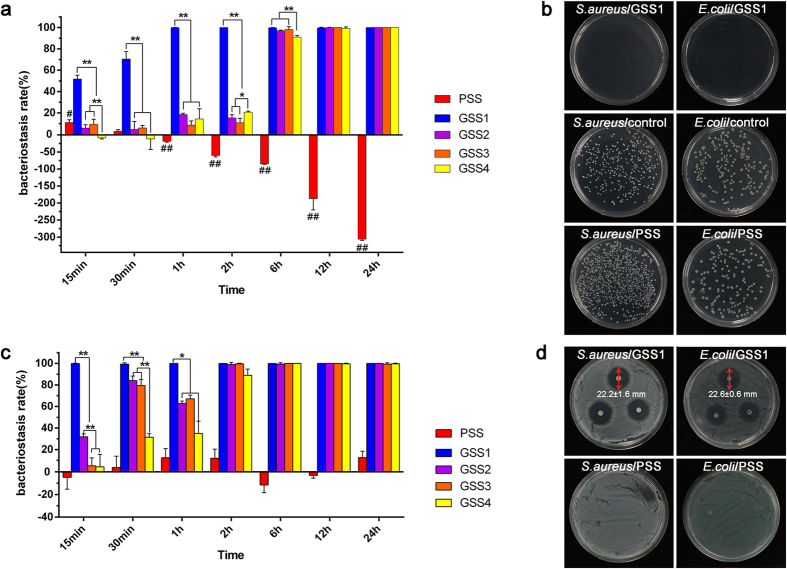
Antimicrobial activity of various GSS samples and PSS against *S. aureus* and *E. coli*. Bacteriostasis rates of various GSS samples and PSS after being incubated with *S. aureus* (**a**) and *E. coli* (**c**) for different durations. (**b**) Variable numbers of colonies in the presence of the PSS, GSS1 or blank and cocultured for 24 h against *S. aureus* and *E. coli*. (**d**) Inhibition zone after coculturing for 24 h (red arrows indicate the diameter of the inhibition zone, n = 3, * and ^#^*p* < 0.05, ** and ^##^*p* < 0.01).

The inhibition zone of GSS1, shown in (Fig. 5d), visually illustrates the high antibacterial activity against *S. aureus* and *E. coli*, with diameters measuring 22.2 ± 1.6 mm and 22.6 ± 0.6 mm, respectively. The PSS showed no antimicrobial effect or a total overgrowth of bacteria after incubation. In addition, we conducted this test in bacterial suspensions of *S. aureus* and *E. coli*, and these results (Figure S2), shown in the supplementary information, agree with inhibition zone and bacterial count results.

#### Bacterial counts after various incubation times

Figure 5 shows the antibacterial activity of the GSS and PSS after being incubated with *S. aureus* and *E. coli* for different durations. The various GSS samples had high antimicrobial activity after 6 h, showing an approximate 100% rate of bacteriostasis for both *S. aureus* and *E. coli* (Fig. 5a,c). Figure 5b shows that neither *S. aureus* nor *E. coli* was present on the culture plates of GSS after 24 h. Figure 5a shows that the bacteriostasis rates of GSS1 against *S. aureus* were 51.7 ± 3.6% and 99.9 ± 0.2% at 15 min and 1 h, respectively. However, in the first 2 h, the bacteriostasis rates of GSS2, GSS3 and GSS4 against *S. aureus* were below 20%. As shown in (Fig. 5c), the bacteriostasis rate of GSS1 against *E. coli* at 15 min was 100%, indicating that *E. coli* was more sensitive to GSS than *S. aureus*. However, for GSS2-4, the time at which the bacteriostasis rate of *E. coli* reached 100% was 2 h. The difference in bacteriostasis rates before 2 h among the various GSS samples indicates that GSS1 was more effective at inhibiting the growth of *S. aureus* and *E. coli* due to the higher concentration of gentamicin, which is consistent with the pharmacological action of gentamicin. This improvement makes the treatment of open fractures with a biodegradable fixation device possible.

As shown in (Fig. 5a), the bacteriostasis rates of PSS against *S. aureus* at 1 h, 2 h, 6 h, 12 h and 24 h were −18.4 ± 2.4%, −60.2 ± 4.2%, −86.9 ± 1.3%, −187.0 ± 32.9% and −306.0 ± 3.6%, respectively. Figure 5b shows that the number of *S. aureus* colonies on the PSS culture plates after 24 h was much greater than on the control plates. This finding suggests that PSS promotes the growth of *S. aureus*.

#### Number and morphology of bacteria on samples

**Figure 6:**
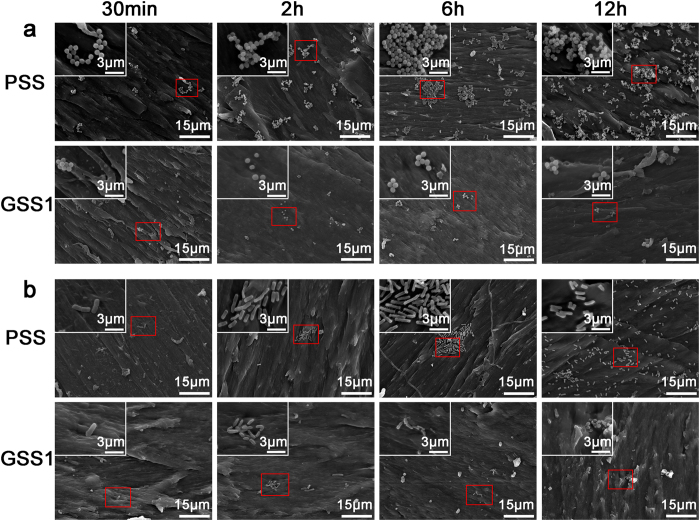
SEM number and morphology of *S. aureus* (**a**) and *E. coli* (**b**) in the presence of PSS and GSS1 for different durations.

SEM was utilized to observe the number and morphology of *S. aureus* (Fig. 6a) and *E. coli* (Fig. 6b) in the presence of PSS and GSS1 for different durations. The number of *S. aureus* strains on PSS increased quickly with prolongation of the incubation time, and the samples were covered with *S. aureus* after 12 h. In contrast, only a few *S. aureus* strains were observed on GSS1 during the first 12 h (Fig. 6a). Similar results were obtained for *E. coli*, as shown in (Fig. 6b). The large reduction in the numbers of *S. aureus* and *E. coli* on GSS1 suggests the high antimicrobial activity of GSS1, which agrees with the counting results shown in (Fig. 5). The *E. coli* present on the PSS had mostly rod shapes (Fig. 6b), with some possessing flagellum, whereas the *E. coli* on GSS1 was corrugated with a distorted shape. Even lysed bacteria with spherical shapes were observed after 12 h (Fig. 6b). In addition, the morphologies of *S. aureus* on the PSS and GSS1 consisted of a similar spherical shape, which also indicates that *E. coli* was more sensitive to GSS1. According to the SEM results, GSS1 can prevent bacterial adhesion and the formation of biofilms on the sample surface.

#### Antimicrobial durability

**Figure 7:**
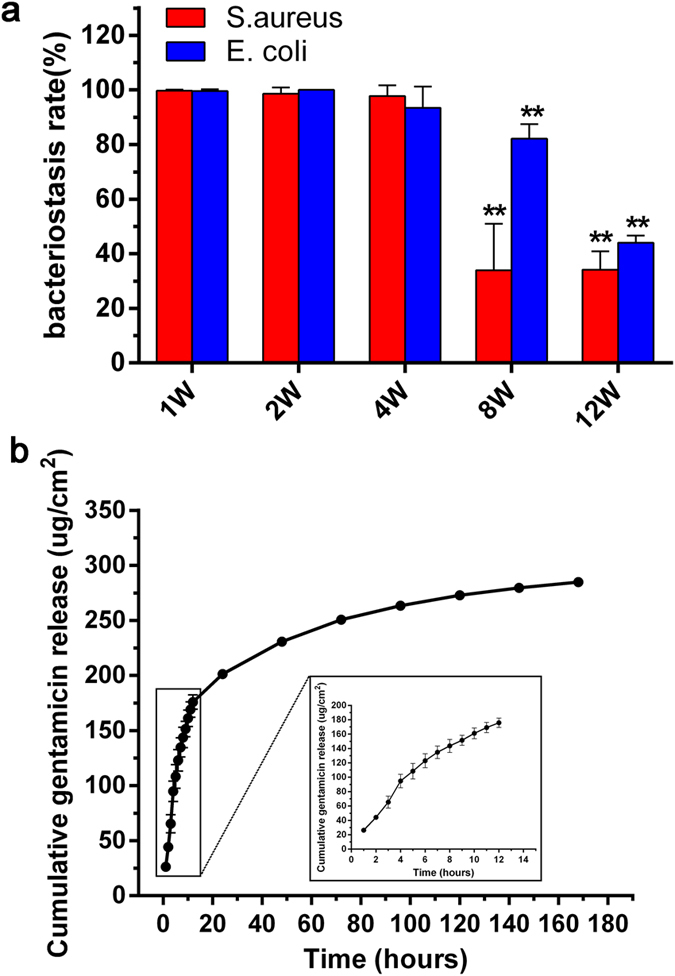
Antibacterial durability of GSS1 against *S. aureus* and *E. coli*. (**a**) Bacteriostasis rate of samples immersed in protease XIV (1 mg/ml) for different durations. (**b**) Cumulative release of gentamicin from samples incubated in PBS at 37 °C for 1 h, 2 h, 3 h, 4 h, 5 h, 6 h, 7 h, 8 h, 9 h, 10 h, 11 h, 12 h, 24 h, 48 h, 72 h, 96 h, 120 h, 144 h and 168 h (n = 3, **p < 0.01).

To further investigate the antimicrobial durability of GSS1, the antibacterial activity of GSS1 after immersion in protease XIV (1 mg/ml) for different durations was assayed, and the results are displayed in (Fig. 7a). After 4 weeks, no significant decrease was observed in the antimicrobial activity of GSS1, which still possessed bacteriostasis rates of 97.8 ± 3.9% and 93.4 ± 7.9% against *S. aureus* and *E. coli*, respectively. After 8 weeks of immersion, the bacteriostasis rate against *S. aureus* and *E. coli* decreased significantly (*p* < 0.01), and 34.1 ± 6.7% and 44.1 ± 2.6% of the bacteriostasis rate was retained after 12 weeks. This result suggests that GSS1 can maintain a high rate of antibacterial activity against *S. aureus* and *E. coli* for at least 4 weeks, which is important for preventing infection in the fracture healing process. We believe that the duration of antibacterial activity *in vivo* should be longer, which is related to the uniform dispersion of gentamicin in the silk protein such that gentamicin will be distributed in the surrounding tissue as the silk degrades. Preventing chronic osteomyelitis, an important and difficult orthopedic and clinical problem, is essential. Therefore, the release of gentamicin from GSS1 in PBS was also investigated, and the results are shown in (Fig. 7b). The release profile shown is divided into two parts: an initial burst release in the initial 12 h due to the rapid dissolution of the gentamicin bound to the surface of the silk (Fig. 1f) and a continuous-phase release for the remaining period of time due to the slow release of the gentamicin inlaid in the silk protein. If the screws are implanted in the body, the gentamicin will be released continuously as the silk degrades. Moreover, the gentamicin immersed in solution indicates that the active component of the bacterial inhibition by GSS was gentamicin.

## Discussion

An internal fixation device is one of the most important risk factors increasing the susceptibility to infections, especially for resorbable materials^38^,^39^. Implant-associated infections and retained implants frequently result in a second surgical procedure to remove the implants^40^, which has substantial economic implications as well as possible time off work required for postoperative recovery^3^. The objective of the present study was to produce an antibacterial and absorbable fixation material with potential to solve this difficult clinical problem by simply combining gentamicin and silk. In the past several decades, many studies have focused on antibacterial silk-based materials to prevent infections. The following three aspects led us to select gentamicin. Foremost, we observed that gentamicin sulfate can dissolve in silk solution uniformly as a result of its good solubility in water^41^. Second, most clinical isolates of *S. aureus* and gram-negative rods that are thought to be mainly responsible for implant-associated infections in orthopedic surgery are sensitive to gentamicin^42^. In addition, gentamicin is one of the few thermostable antibiotics^41^,^43^.

In this study, gentamicin sulfate was successfully incorporated within silk-based screws by physical dissolution in HFIP. The presence of the gentamicin particles endowed GSS with high and durable antimicrobial activity. On the one hand, bacterial contamination during surgery, via air or direct contact and subsequent bacterial adhesion onto the biomaterial surface, is the crucial initial step in the pathogenesis of implant-associated infections^44^. GSS1 completely inhibited the growth of *S. aureus* and *E. coli* within 1 h, whereas 6 h was required in other groups (GSS2, GSS3, GSS4) (Fig. 5a,c). Hence, the rapid and high bactericidal effect of GSS is critical for reducing the risk of implant-associated infection during the perioperative period. On the other hand, the GSS are intended to provide locally sufficient drug levels while maintaining low systemic levels to avoid the risk of organ toxicity, such as hearing or kidney damage, and resistant strains^45^. Therefore, 16 mg of gentamicin incorporated per g of silk (GSS1) is the most suitable concentration.

In addition, the decrease in antibacterial activity after 4 weeks was likely due to an insufficient amount of gentamicin remaining in the sample to completely inhibit the bacteria. We believe that the duration of antibacterial activity *in vivo* should be longer with the gentamicin distributed in the surrounding tissue as the silk degrades. Tissue damage caused by surgery and foreign body implantation further increases the susceptibility to infections in the process of fracture healing^38^. Therefore, the durable antimicrobial activity of GSS1 is important in preventing chronic infection to avoid implant failures or a second surgery. Interestingly, we found that PSS may promote the growth of *S. aureus*, which will increase the risk of implant-associated infection. This behavior was possibly due to the rough surface and degradation products of the PSS. Therefore, antibacterial activity must be conferred to silk fibroin by loading with gentamicin. Furthermore, local antimicrobial prophylaxis may carry a reduced risk of inducing resistant strains than the systemic therapy used in orthopedic implant surgery^17^.

Remarkable mechanical properties and excellent biocompatibility are prerequisites for loadbearing biomedical implants, especially for an internal fixation device based on polymer biomaterials^46^. It has been shown that silk fibroin is such a natural biopolymer with high mechanical strength, biodegradability and excellent biocompatibility^21^,^22^. However, it is unclear whether these basic features of silk will be altered after combining silk with gentamicin sulfate. Our research indicates that GSS maintains the remarkable mechanical properties and excellent biocompatibility exhibited by PSS. Bending and shear forces are two of the most prominent forces for fixation pin function^47^. The results of the three-point bending test and double-shear test demonstrated that GSS have impressive mechanical features, similar to those of PSS, sufficient to withstand loading during bone formation. Moreover, GSS and PSS have Young’s modulus values of approximately 8 GPa compared with approximately 18 GPa for cortical bone^48^,^49^. However, the Young’s modulus of Ti-6Al-4V is 113.8 GPa, which can result in stress shielding during fracture healing^50^. It has been reported that more flexible and absorbable fixation devices make bone healing faster and more complete^51^. In addition, the results of the CCK-8 assay and flow cytometry analysis indicate that there were no detrimental effects on cell proliferation or apoptosis from the PSS or GSS1. Thus, GSS exhibited good biocompatibility, similar to that of PSS, whose excellent biocompatibility was reported in previous studies^21^,^52^. Therefore, incorporating gentamicin sulfate does not change the biocompatibility of silk protein.

The degradation of PSS was consistent with a second-order polynomic trendline (Fig. 2e), which agrees with previous studies^21^,^37^. Interestingly, the degradation rate of GSS1 was apparently faster than that of PSS and was consistent with a third-order polynomic trendline (Fig. 2e). One explanation for this difference may be associated with the voids that increased the surface area exposed to the enzyme (Fig. 2b,d). We may assume that these voids are due to the dissolution of gentamicin particles, resulting in a porous structure because the sizes of the voids were similar to those of the gentamicin particles. If this is the case, the degradation rate of GSS may be tunable by changing the number and size of the gentamicin particles. Furthermore, a combination of the surface roughness caused by the rapid degradation of GSS1 and porosity favored human bone marrow-derived mesenchymal stem cell differentiation toward bone-like tissue^46^. In addition, rapid degradation and bone-like tissue are beneficial to a gradual transfer of the load-bearing burden to the developing tissue during fracture healing, which supports the restoration and maintenance of tissue function over the life of the patient^37^. Generally, after approximately 3 months, bone union has gained enough strength and rigidity^53^, corresponding to the time at which the GSS were approximately 60% of the mass remaining *in vitro*. We deduced that the PSS samples would completely degrade *in vitro* after approximately 8 months according to the degradation data, and the time required for GSS1 to completely degrade is approximately 5 months. However, these approximations cannot directly represent degradation *in vivo* due to the complex environment in the body, such as the presence of various enzymes and cell types. In the future, research on *in vivo* degradation will need to be explored. We believe that GSS can be an optimal fixation material whose initial strength can provide excellent fixation with a mass loss profile that is suitable for the bone-healing process.

The rapid swelling of the silk screws results in a reduction in mechanical strength, which may cause the implantation process to fail^21^. In addition, the increase in the diameter of the screws during the operation will make it difficult for the surgeon to implant the screws. Therefore, the decreased water-absorbing capacity of GSS1 in (Fig. 3) is beneficial to the implantation of the screw by the surgeon. For at least 30 min, the diameter of GSS1 did not increase significantly when it came into contact with water in the body, which is a sufficient time for the surgeon to implant the screw.

However, the limitation of these GSS is the size of the screws. When the sizes of the molds were increased to increase the diameter of the blanks and the screws, there were bubbles in the silk blanks that would affect the functioning and mechanical stability of the screws^21^. Future research should be conducted to verify the features of GSS *in vivo*, such as their antibacterial ability, regulation of degradation and biocompatibility, and to investigate their detailed mechanism.

## Conclusion

Pure silk-based screws (PSS) promoted the growth of *S. aureus in vitro*. To prevent implant-associated infections after fixation surgery, gentamicin was successfully incorporated into silk-based screws. Gentamicin-loaded silk-based screws (GSS) not only retained the impressive mechanical features and biocompatibility of PSS but also exhibited high and durable antimicrobial activity against *S. aureus* and *E. coli in vitro*. The degradation rate of GSS increased, which was related to the dissolution of gentamicin particles, leaving a porous structure. In addition, the decreased water absorption of GSS will give a surgeon more time to implant this screw. Our findings indicate that this antibacterial silk-based fixation material can overcome the limitations of metal and traditionally resorbable devices, with great potential for use in orthopedic implants to reduce the incidence of second surgical procedures for a given clinical application.

## Additional Information

**How to cite this article**: Shi, C. *et al.* An antibacterial and absorbable silk-based fixation material with impressive mechanical properties and biocompatibility. *Sci. Rep.* **6**, 37418; doi: 10.1038/srep37418 (2016).

**Publisher’s note:** Springer Nature remains neutral with regard to jurisdictional claims in published maps and institutional affiliations.

## Supplementary Material

Supplementary Information

## Figures and Tables

**Table 1:**
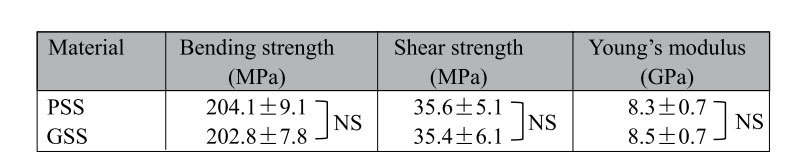
Mechanical properties of PSS and GSS samples (length, 17 mm, and diameter, 5 mm).

The data are expressed as the mean ± SD (n = 5); NS indicates no significant differences.
