# Supplementary material for: An antibacterial and absorbable silk-based fixation material with impressive mechanical properties and biocompatibility
Source: Sci Rep. 2016 Nov 21;6:37418. doi: 10.1038/srep37418 (PMC5116670; doi:10.1038/srep37418)
Supplement: Supplementary Information [file srep37418-s1.pdf]

An antibacterial and absorbable silk-based fixation material with impressive mechanical properties and biocompatibility

Chenglong Shi, Xiaobing Pu, Guan Zheng, Xinglong Feng, Xuan Yang, Baoliang Zhang, Yu Zhang, Qingshui Yin, Hong Xia

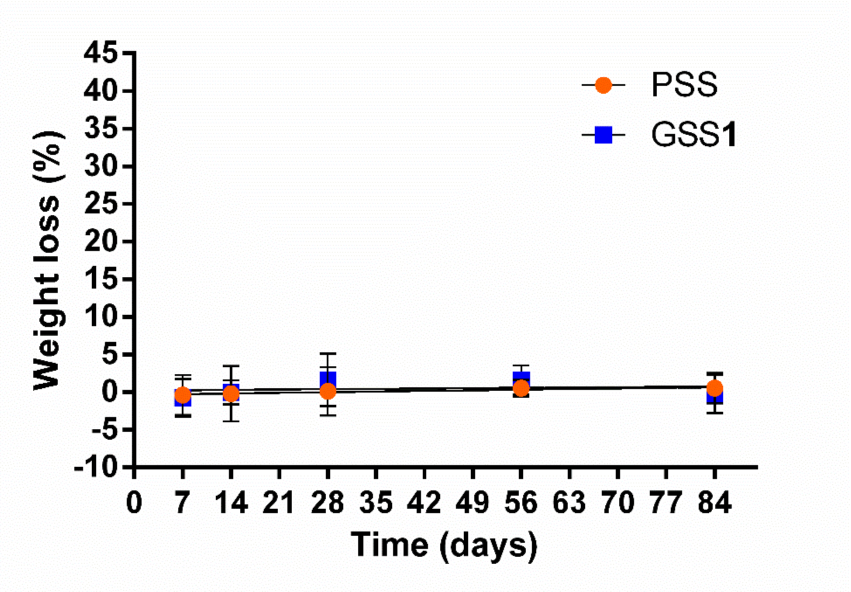

Figure-S1 Weight loss of PSS and GSS1 after immersion in PBS for different durations.

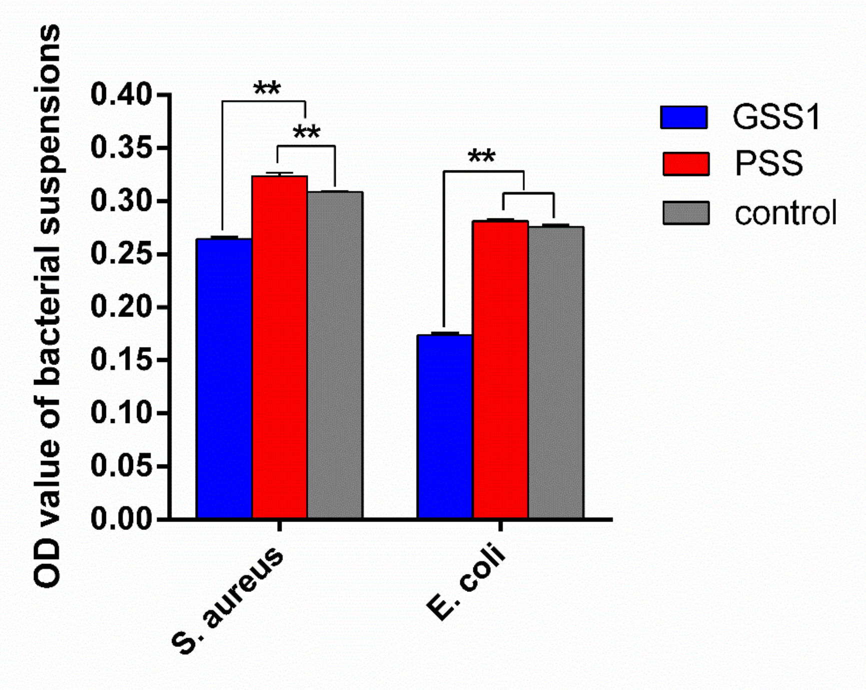

Figure-S2 OD values of S. aureus and E. coli suspensions after 24 h of incubation. After 24 h of incubation, the OD values of the S. aureus and E. coli suspensions in GSS1 were significantly (p<0.01) lower than in the control group. In addition, the OD values of S. aureus in the PSS group were significantly (p<0.01) higher than in the control group. However, the

OD values of E. coli between the PSS and control groups were not significantly ($p>0.05$) different (** $p<0.01$).
